# Sugemalimab plus chemotherapy vs. chemotherapy for treatment of Chinese patients with esophageal squamous cell carcinoma: a cost effectiveness analysis to inform decision making

**DOI:** 10.3389/fonc.2025.1459695

**Published:** 2025-06-05

**Authors:** Qiuping Chen, Quan Sun, Baixue Li

**Affiliations:** ^1^ School of Basic Medical Sciences, Chengdu University of Traditional Chinese Medicine, Chengdu, Sichuan, China; ^2^ Health Care Security and Pharmacoeconomic Evaluation Branch, China Medical Education Association, Beijing, China; ^3^ Department of Health Economics and Outcome Research, Hainan Zhongwei Institute of Health Economy Development, Haikou, Hainan, China

**Keywords:** cost-effectiveness, sugemalimab, esophageal squamous cell carcinoma, partitioned survival model, PD-L1

## Abstract

**Background:**

The GEMSTONE-304 trial established the clinical benefits of sugemalimab plus chemotherapy in advanced esophageal squamous-cell carcinoma (ESCC). This study evaluates the cost-effectiveness of this regimen versus chemotherapy alone as the first-line treatment for advanced ESCC patients from the perspective of China’s health system.

**Methods:**

We established a partitioned survival model based on GEMSTONE-304 trial data, we simulated lifetime outcomes through three health states: progression-free survival, progressive disease, and death. Key parameters included quality-adjusted life years (QALYs) and incremental cost-effectiveness ratio (ICER), analyzed with 5% discounting. Sensitivity analyses encompassed probabilistic, one-way, and scenario evaluations.

**Results:**

The sugemalimab combination yielded 0.336 incremental QALYs at $ 44,182.03 additional cost (ICER = $ 131,544.70/QALY). PD-L1 subgroup ICERs exhibited dose-dependent efficacy: $ 187,421.63/QALY (Combined Positive Score (CPS) < 1), $ 175,689.56 (1 ≤ CPS < 10), and $ 130,349.21 (CPS ≥ 10). Scenario analysis demonstrated ICER reduction to $ 51,454.12/QALY under consideration of patient assistance program. None of the results demonstrated cost-effectiveness for this therapeutic regimen. Sensitivity analyses identified sugemalimab pricing as the dominant driver of ICER, while simultaneously validating the model’s internal and external validity. Price cap simulations determined that a minimum 91.20% price reduction is required to achieve cost-effectiveness.

**Conclusion:**

Current pricing renders sugemalimab combination therapy economically unfavorable as first-line ESCC treatment in China. Strategic price adjustments could enhance cost-effectiveness potential.

## 1 Introduction

Esophageal cancer remains a major global health challenge (1, 2), ranking as the seventh most common malignancy in Chinese females and fifth in males, with approximately 224,000 new cases and 187,500 deaths annually (3). Histologically classified into esophageal adenocarcinoma (EAC) and esophageal squamous cell carcinoma (ESCC) (4, 5), the latter accounts for 80% of cases and presents particular therapeutic challenges (6). The absence of effective early markers and screening modalities often leads to late diagnosis (stages II or III), missing optimal treatment windows (7). Current first-line chemotherapy regimens (cisplatin/5-fluorouracil, paclitaxel/carboplatin (8)) demonstrate limited efficacy (9, 10), contributing to a dismal 5-year survival rate of 18% (11).

In recent years, immunotherapies targeting PD-1 (12–14) and PD-L1 (15–17) have significantly advanced ESCC treatment. Landmark trials including KEYNOTE-181 (pembrolizumab), ORIENT-15 (sintilimab), and CheckMate 648 (nivolumab) have demonstrated significant survival benefits in advanced ESCC, establishing immunotherapy as a first-line standard (18–20). Sugemalimab, a fully humanized PD-L1 monoclonal antibody (21),, binds to the PD-1 receptor and blocks the PD-1/PD-L1 interaction, thereby enhancing anti-tumor immune responses (22, 23). It has demonstrated good efficacy and safety in treating metastatic non-small-cell lung cancer (NSCLC) (24, 25) and relapsed or refractory extranodal natural killer/T-cell lymphoma (26). The GEMSTONE-304 trial first demonstrated that sugemalimab combined with chemotherapy significantly improves median overall survival (OS) (15.3 vs 11.5 months) and progression-free survival (PFS) (6.2 vs 5.4 months) in advanced ESCC (27). Notably, despite these clinical benefits, the high cost of sugemalimab necessitates a cost-effectiveness analysis to assess whether the incremental costs justify the survival benefits, crucial for optimizing healthcare resource utilization.

This study innovatively constructs a three-state partitioned survival model (PSM) (derived from GEMSTONE-304 data (28)) to evaluate long-term cost-effectiveness from the Chinese health system perspective. The analysis not only fills a critical gap in China’s immunotherapy economic evaluations but also provides pivotal evidence for dynamic China’s National Reimbursement Drug List (NRDL) adjustments and optimal allocation of cancer control resources, carrying significant policy implications for refining China’s precision medicine value assessment framework.

## 2 Materials and methods

### 2.1 Population

The study cohort was derived from the GEMSTONE-304 phase III trial (NCT04187352) involving 540 patients with unresectable locally advanced esophageal squamous cell carcinoma (27). Inclusion criteria comprised: 18–75 years old, ECOG PS 0-1, life expectancy ≥ 3 months, measurable lesions per RECIST 1.1, and adequate organ function. Exclusion criteria excluded non-ESCC histology, active central nervous system (CNS) metastases or carcinomatous meningitis, prior systemic therapy, PD-1/L1 inhibitor exposure, Human Immunodeficiency Virus/Acquired Immune Deficiency Syndrome tests, organ transplantation history, and concurrent malignancies (29). Patients were randomized to either sugemalimab plus chemotherapy (n=358) or chemotherapy alone (n=182). The intervention group received sugemalimab (1200 mg iv q3w) with cisplatin (80 mg/m^2^ iv d1) and 5-FU (800 mg/m^2^ iv d1–4 q3w), while the control group received identical chemotherapy regimens without sugemalimab. This therapeutic alignment ensured comparability between groups, with treatment continuation until disease progression or intolerable toxicity.

The model incorporated demographic parameters from the Chinese population, including an average body weight of 65 kg and body surface area of 1.72 m^2^ (29) All patients initiated first-line therapy until disease progression or intolerable toxicity, with subsequent second-line treatment continuing until death. As the GEMSTONE-304 trial did not specify second-line regimens, we adopted the Chinese Society of Clinical Oncology Guidelines for the Diagnosis and Treatment of Esophageal Cancer 2024 (CSCO 2024) guideline-recommended protocol using irinotecan hydrochloride injection (350 mg/m^2^ q3w) combined with tegafur/gimeracil/oteracil (S-1) capsules (60 mg bid) for both treatment arms to maintain model stability (30). This standardized approach mitigated potential bias from differential second-line therapies while reflecting current clinical practice patterns in China.

### 2.2 Model construction

**Figure 1:**
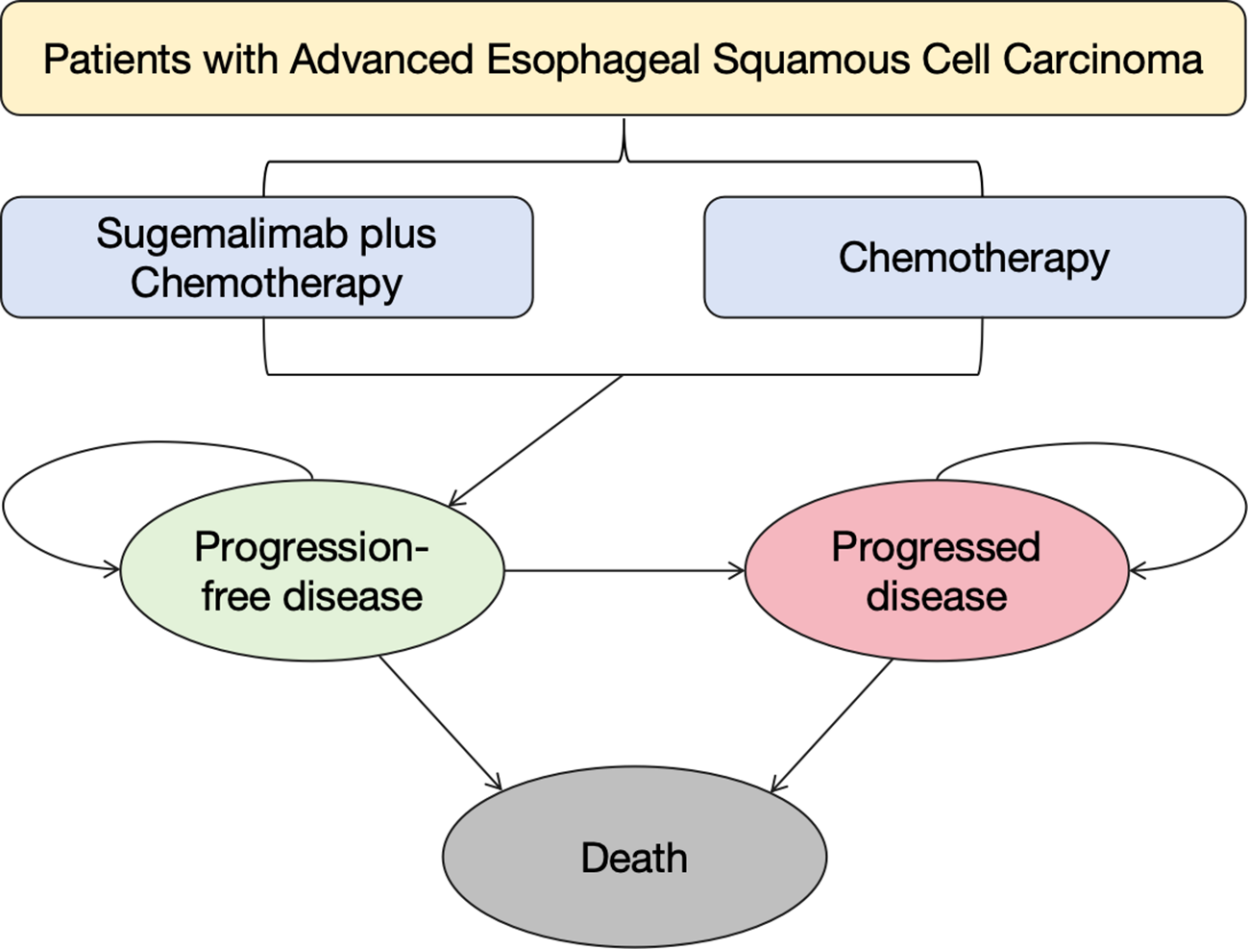
Patient health state transition matrix.

A PSM was developed to simulate disease progression in ESCC patients, incorporating three mutually exclusive health states: PFS, progressive disease (PD), and death (31). The model’s 3-week cycle length were aligned with the trial’s interval design, ensuring temporal consistency between intervention phases, and 10-year time horizon were calibrated to Chinese ESCC epidemiological data (5-year survival <18%) (11). All patients initiated in the PFS state, with transition probabilities between states derived from trial survival curves (**Figure 1**). The homogeneous baseline characteristics of trial participants (mean age 62.75 years, no baseline progression) enabled standardized state transition modeling. Cost and quality-adjusted life year (QALY) outcomes were discounted at 5% annually following the latest Chinese pharmacoeconomic guideline (32). This temporal framework captured both short-term treatment effects and long-term survival patterns, while the cycle length alignment with clinical trial intervals ensured therapeutic exposure accuracy.

### 2.3 Survival analysis

**Figure 2:**
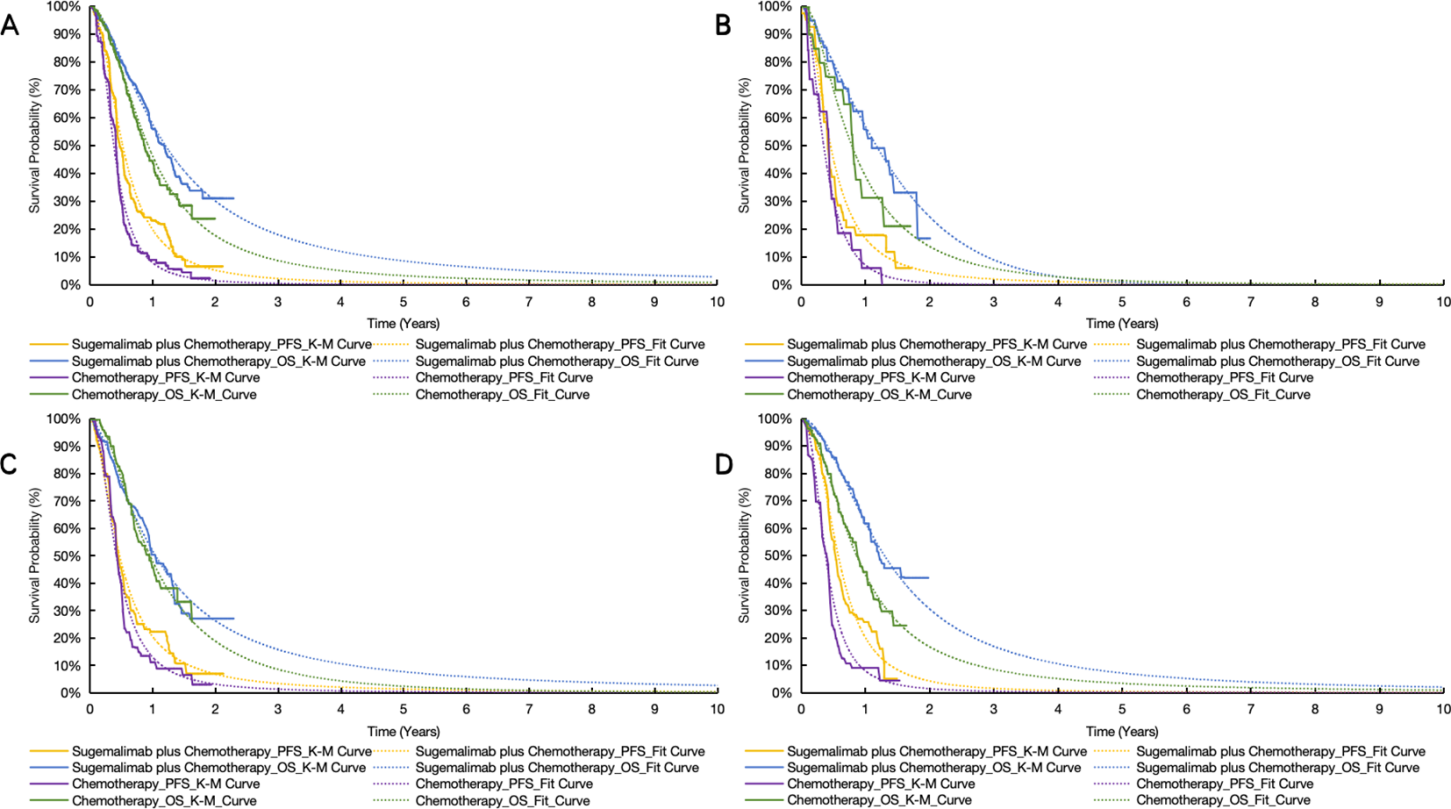
Original and reconstructed survival curves. **(A)** the whole patient. **(B)** patients with PD-L1 CPS < 1. **(C)** patients with PD-L1 1 < CPS < 10. **(D)** patients with PD-L1 CPS ≥ 10. OS indicates overall survival; PFS, progression-free survival; S + C, sugemalimab plus chemotherapy.

Survival data from the GEMSTONE-304 trial were extracted using GetData Graph Digitizer (33) and reconstructed in R software using the packages ‘survival’, ‘survHE’, and ‘survminer’. Six parametric distributions (Exponential, Weibull, Log-logistic, Log-normal, Gompertz and Generalized Gamma) were evaluated for PFS and OS curve fitting through maximum likelihood estimation (34). The optimal parametric models (**Table 1**) were selected based on statistical criteria (Akaike/Bayesian information criteria minimization) combined with visual examination of curve alignment, with detailed parameters documented in **eTable 1**. This methodology enabled extrapolation of trial survival outcomes to the 10-year model horizon, with cycle-specific health state occupancy proportions mathematically derived from the reconstructed survival curves (**Figure 2**).

**Table 1:** Fitting function and parameters of survival curve of each scenario.

Group	Scenario	States	Distribution	Parameter 1		Parameter 2		AIC	BIC
Sugemalimab plus Chemotherapy	The whole patient	PFS	Log-logistic	9.06	(Shape)	2.15	(Scale)	1633.05	1640.81
		OS	Log-logistic	20.92	(Shape)	1.66	(Shape)	1442.43	1450.19
	PD-L1 CPS<1	PFS	Log-logistic	7.92	(Shape)	2.05	(Scale)	212.24	215.66
		OS	Weibull	0.01	(Meanlog)	1.32	(Sdlog)	187.77	190.60
	PD-L1 1<CPS<10	PFS	Log-logistic	8.21	(Shape)	1.82	(Scale)	770.10	776.29
		OS	Log-logistic	18.14	(Meanlog)	1.59	(Sdlog)	710.07	717.03
	PD-L1 CPS≥10	PFS	Log-logistic	10.00	(Shape)	2.50	(Scale)	672.95	679.02
		OS	Log-logistic	22.51	(Meanlog)	1.90	(Sdlog)	552.15	558.22
Chemotherapy	The whole patient	PFS	Log-logistic	6.61	(Shape)	2.53	(Scale)	791.57	797.97
		OS	Log-logistic	16.12	(Shape)	2.00	(Scale)	779.32	785.73
	PD-L1 CPS<1	PFS	Log-normal	1.80	(Meanlog)	0.72	(Sdlog)	94.36	96.45
		OS	Log-normal	2.62	(Meanlog)	0.86	(Sdlog)	104.25	106.34
	PD-L1 1<CPS<10	PFS	Log-logistic	7.32	(Shape)	2.20	(Scale)	364.43	369.27
		OS	Log-normal	2.81	(Meanlog)	0.83	(Sdlog)	340.86	345.70
	PD-L1 CPS≥10	PFS	Log-logistic	6.62	(Shape)	2.54	(Scale)	338.32	343.06
		OS	Log-logistic	14.96	(Shape)	1.90	(Scale)	339.34	344.06

PD-L1, programmed death ligand-1; CPS, combined positive score; PFS, progression-free survival; OS, overall survival; AIC, Akaike information criterion; BIC, Bayesian information criterion.

### 2.4 Cost and utility estimates

The cost-effectiveness analysis adopted the Chinese healthcare system perspective, incorporating direct medical costs encompassing drug acquisition, adverse event (AE) management, hospitalization, and progressive disease treatment. Drug costs for sugemalimab, cisplatin, 5-FU and camrelizumab were obtained from the Chinese health industry’s big data service platform (https://db.yaozh.com/), with AE-related expenses (grade ≥ 3 incidence ≥ 3%) and hospitalization fees derived from published literature (35–38). Specifically, severe hematologic toxicities (anemia, neutropenia, thrombocytopenia) and gastrointestinal events (nausea, vomiting) were included based on GEMSTONE-304 safety profiles (36–38). Health state utilities were derived from international ESCC studies due to lack of Chinese-specific data (39, 40). The willingness-to-pay (WTP) threshold was established at 1.94 times China’s 2024 per capita GDP ($ 23901.90/QALY), aligned with national guideline and community-based surveys (32, 41). All relevant inputs are presented in **Table 2**. According to the consumer price index (CPI) of the National Bureau of Statistics of China, all the cost parameters involved in this study are converted to 2023 (the latest consumer price index results released by China).

**Table 2:** Model parameters and the range of the sensitivity analysis.

Variable	Baseline value	Lower	Upper	Distribution	Source
Cost of drugs ($)^a^					
Sugemalimab	1732.5 (600mg)	1386	2079	gamma	http://db.yaozh.com
Cisplatin	2.68 (30mg)	2.14	3.22	gamma	http://db.yaozh.com
Fluorouracil	20.44 (250mg)	16.35	24.53	gamma	http://db.yaozh.com
Cost after disease progression^b^	753.49 (per cycle)	602.79	904.19	gamma	http://db.yaozh.com
Hospitalization expense (Cycle)	142.10	15.89	23.83	gamma	(37)
Cost of AEs ($)^a^					
Nausea	82.62	66.10	99.14	gamma	(38)
Anemia	140.40	112.32	168.48	gamma	(36)
Neutrophil count decreased	116.37	93.10	139.64	gamma	(36)
White blood cell count decreased	116.37	93.10	139.64	gamma	(36)
Vomiting	82.62	66.10	99.14	gamma	(38)
Platelet count decreased	1523.82	1219.06	1828.58	gamma	(36)
Lymphocyte count decreased	149.60	119.68	179.52	gamma	(39)
Risk of AEs					
Sugemalimab plus Chemotherapy group					
Nausea	3.7%	2.96%	4.44%	beta	GEMSTONE-304
Anemia	16.7%	13.36%	20.04%	beta	GEMSTONE-304
Neutrophil count decreased	20.4%	16.32%	24.48%	beta	GEMSTONE-304
White blood cell count decreased	8.8%	7.04%	10.56%	beta	GEMSTONE-304
Vomiting	5.1%	4.08%	6.12%	beta	GEMSTONE-304
Platelet count decreased	5.7%	4.56%	6.84%	beta	GEMSTONE-304
Lymphocyte count decreased	4.5%	3.60%	5.40%	beta	GEMSTONE-304
Chemotherapy group					
Nausea	4.9%	3.92%	5.88%	beta	GEMSTONE-304
Anemia	14.3%	11.44%	17.16%	beta	GEMSTONE-304
Neutrophil count decreased	20.9%	16.72%	25.08%	beta	GEMSTONE-304
White blood cell count decreased	10.4%	8.32%	12.48%	beta	GEMSTONE-304
Vomiting	4.9%	3.92%	5.88%	beta	GEMSTONE-304
Platelet count decreased	4.4%	3.52%	5.28%	beta	GEMSTONE-304
Lymphocyte count decreased	3.3%	2.64%	3.96%	beta	GEMSTONE-304
Utility					
Utility of PFS	0.75	0.60	0.90	beta	(40, 41, 44)
Utility of PD	0.60	0.48	0.72	beta	(40, 41, 44)
Others					
Discount rate (%)	5.00	4.00	6.00	beta	(32)

AEs, adverse reactions; PFS, progression-free survival; PD, progressive disease.

a All costs have been adjusted to US dollars in 2023 based on China’s Consumer Price Index (CPI).

b Irinotecan hydrochloride surplus Tegafur/Gimeracil/Oteracil (S-1).

### 2.5 Sensitivity analysis

Sensitivity analyses were systematically conducted to validate model robustness and parameter influence on incremental cost-effectiveness ratios (ICERs). One-way sensitivity analysis (OWSA) examined individual parameter variations by ±20% when literature-based ranges were unavailable, while probabilistic sensitivity analysis (PSA) employed 1,000 Monte Carlo simulations with Gamma distributions for cost parameters and Beta distributions for utilities/AE risks (42). Key drivers of cost-effectiveness were visualized through tornado diagrams, with uncertainty characterized via incremental cost-effectiveness scatterplots and cost-effectiveness acceptability curves (CEACs).

To address limitations in Chinese-specific health utility data, sensitivity analyses incorporated alternative utility estimates from international ESCC studies (43–45), systematically evaluating cross-cultural variations in quality-of-life valuations.

### 2.6 Subgroup and scenario analyses

Subgroup analyses evaluated cost-effectiveness variations across PD-L1 expression subgroups (CPS < 1, 1 ≤ CPS < 10, CPS ≥ 10) using biomarker-stratified survival data (44). Scenario analysis modeled the economic impact of implementing the NSCLC patient assistance program (PAP) for ESCC treatment, assuming hypothetical approval. The PAP structure incorporates three donation phases: initial (2 purchased cycles + 2 free), secondary (2 + 25 cycles), and tertiary (1 + 3 cycles) support tiers. At the current NSCLC price ($1,732.5/600mg), the analysis assumed 100% PAP participation with progressive cost reductions. This dual approach assessed both biological heterogeneity through PD-L1 stratification and financial accessibility via pricing scenarios, maintaining consistent efficacy assumptions across analyses.

## 3 Results

### 3.1 Base–case analysis results

The base-case analysis demonstrated differential economic outcomes across treatment arms (**Table 3**). For the whole patient, sugemalimab combination therapy yielded an incremental 0.336 QALYs at an additional cost of $ 44,182.03 versus chemotherapy alone, resulting in an ICER of $ 131,544.70/QALY, exceeding the predefined WTP threshold. Subgroup analyses revealed biomarker-dependent cost-effectiveness gradients: PD-L1 CPS ≥10 subgroup showed optimal economic performance (ICER = $ 130,349.21/QALY), while CPS <1 subgroup exhibited limited value (ICER = $ 187,421.63/QALY). Scenario analysis incorporating patient assistance programs reduced the ICER to $ 51,454.12/QALY, yet remained above conventional affordability benchmarks. These findings underscore the necessity of strategic price adjustments to align sugemalimab’s cost with health system sustainability requirements.

**Table 3:** Base-case analysis results.

Group	Group	Costs ($) ^a^	QALYs	Incremental costs ($) ^a^	Incremental QALYs	ICER ($/QALY) ^a^
Sugemalimab plus chemotherapy	The whole patient	59,344.19	1.193	44,182.03	0.336	131,544.70
	CPS<1	49,910.74	0.923	37,522.92	0.200	187,421.63
	1<CPS<10	55,461.57	1.102	41,874.51	0.238	175,689.56
	CPS>10	61,178.75	1.187	46,918.00	0.360	130,349.21
	Consideration of PAP	32,308.68	1.193	17,281.94	0.336	51,454.12
Chemotherapy	The whole patient	15,162.17	0.857	——	——	——
	CPS<1	12,387.82	0.723	——	——	——
	1<CPS<10	13,587.06	0.864	——	——	——
	CPS>10	14,260.76	0.827	——	——	——
	Consideration of PAP	15,026.74	0.857	——	——	——

CPS, combined positive score; QALYs, quality-adjusted life years; PAP, patient assistance program; ICER, incremental cost-effectiveness ratio.

a All costs have been adjusted to US dollars in 2023 based on China’s Consumer Price Index (CPI).

### 3.2 Sensitivity analyses

#### 3.2.1 One-way sensitivity analysis

**Figure 3:**
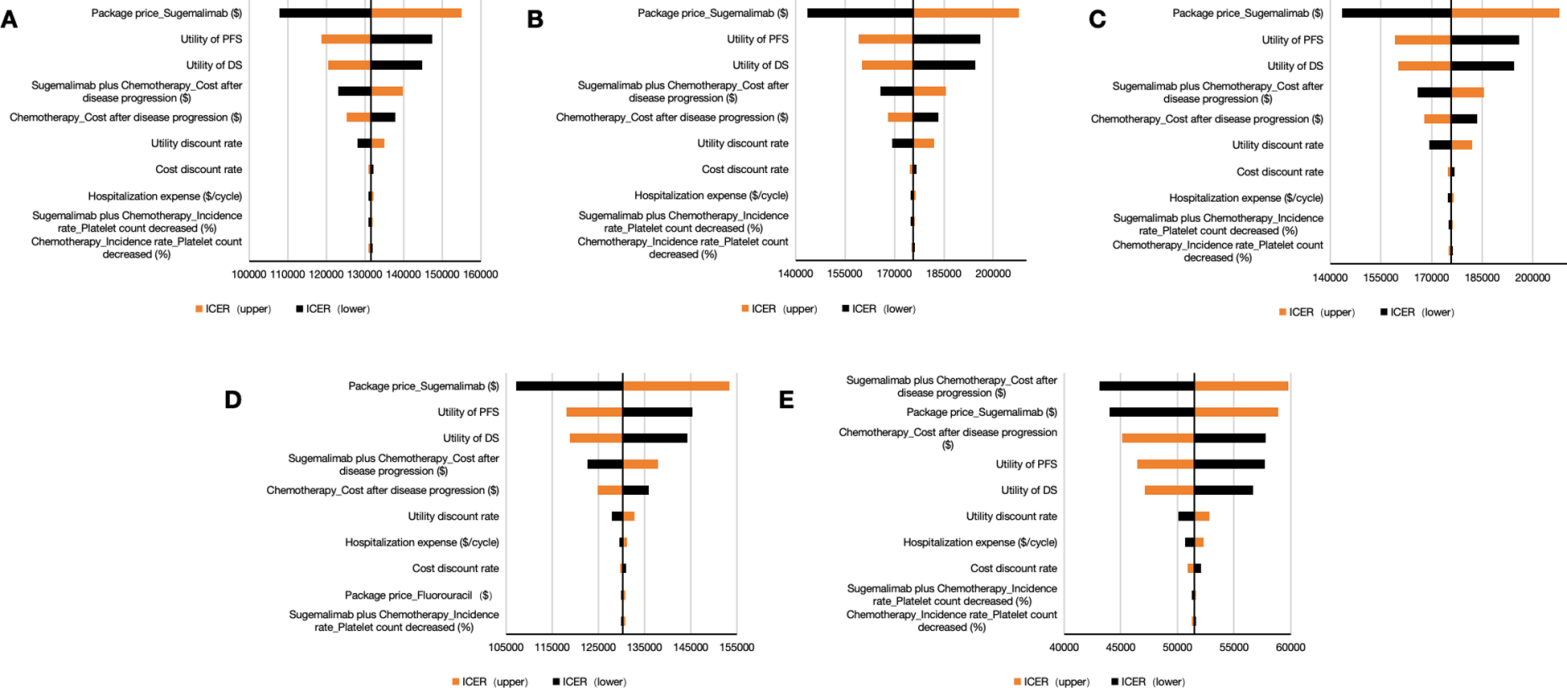
Tornado diagrams for one-way sensitivity analysis. **(A)** the whole patient. **(B)** patients with PD-L1 CPS < 1. **(C)** patients with PD-L1 1 < CPS < 10. **(D)** patients with PD-L1 CPS ≥ 10. **(E)** Consideration of PAP. PFS indicates progression-free survival; PD, progressive disease; S + C, sugemalimab plus chemotherapy; ICER, incremental cost-effectiveness ratio; PAP, patient assistance program.

The OWSA identified sugemalimab’s unit price as the principal ICER determinant across all analytical scenarios (**Figure 3**). For the whole patient, the package price of sugemalimab, utility of PD, and utility of PFS were the factors with the significant influence (**Figure 3A**). Subgroup analyses revealed consistent price sensitivity across difference PD-L1 expression strata (**Figures 3B–D**). Scenario analysis incorporating PAPs demonstrated amplified sensitivity to disease progression costs, highlighting financial vulnerability in late-line treatment phases (**Figure 3E**). These findings collectively establish drug pricing as the critical leverage point for value assessment in China’s reimbursement framework.

#### 3.2.2 Probabilistic sensitivity analysis

**Figure 4:**
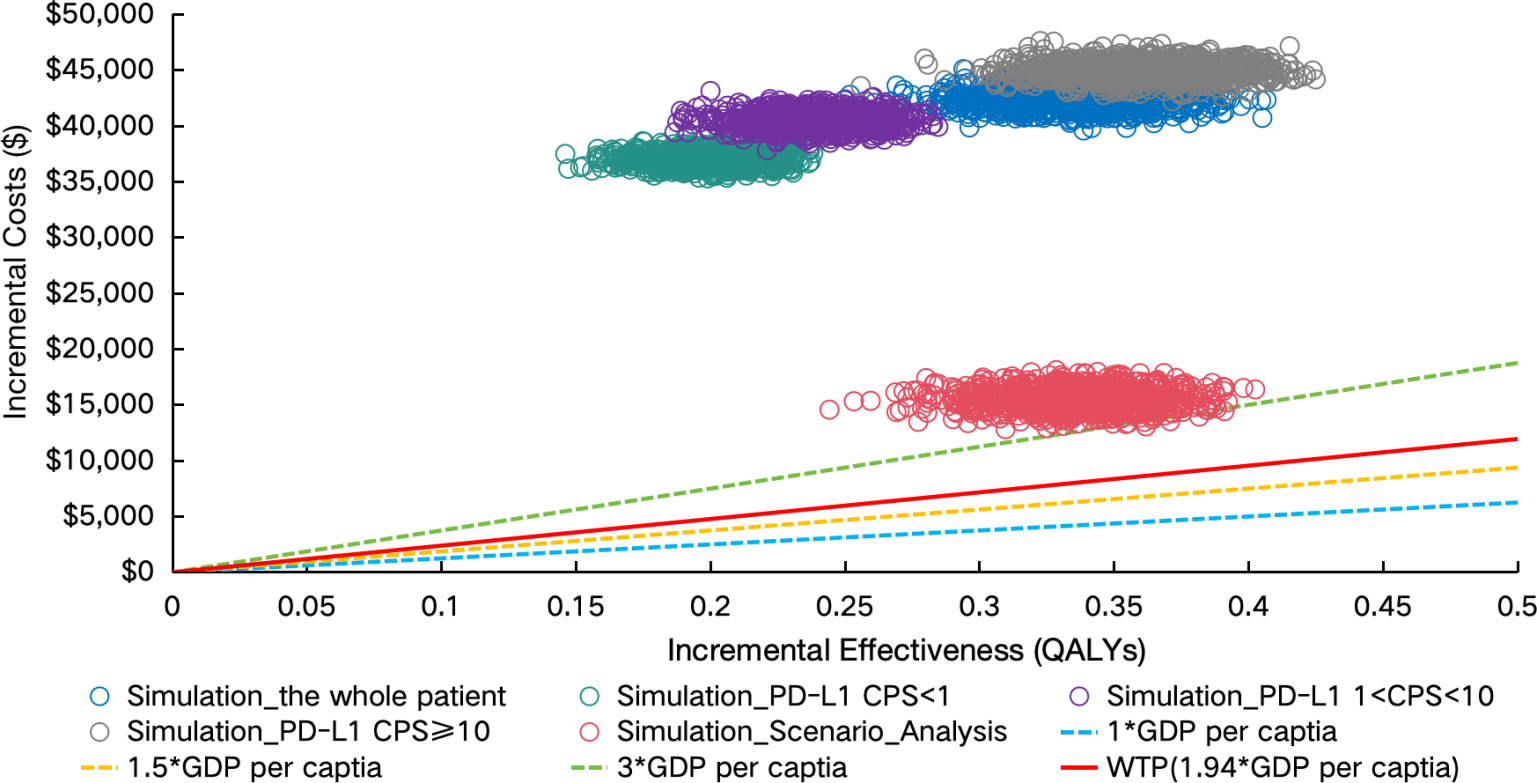
Incremental cost-effectiveness scatter plot. PD-L1 indicates programmed death ligand-1; CPS, combined positive score; GDP, gross domestic product; WTP, willingness to pay; QALYs, Quality-adjusted life years; PAP, patient assistance program.

**Figure 5:**
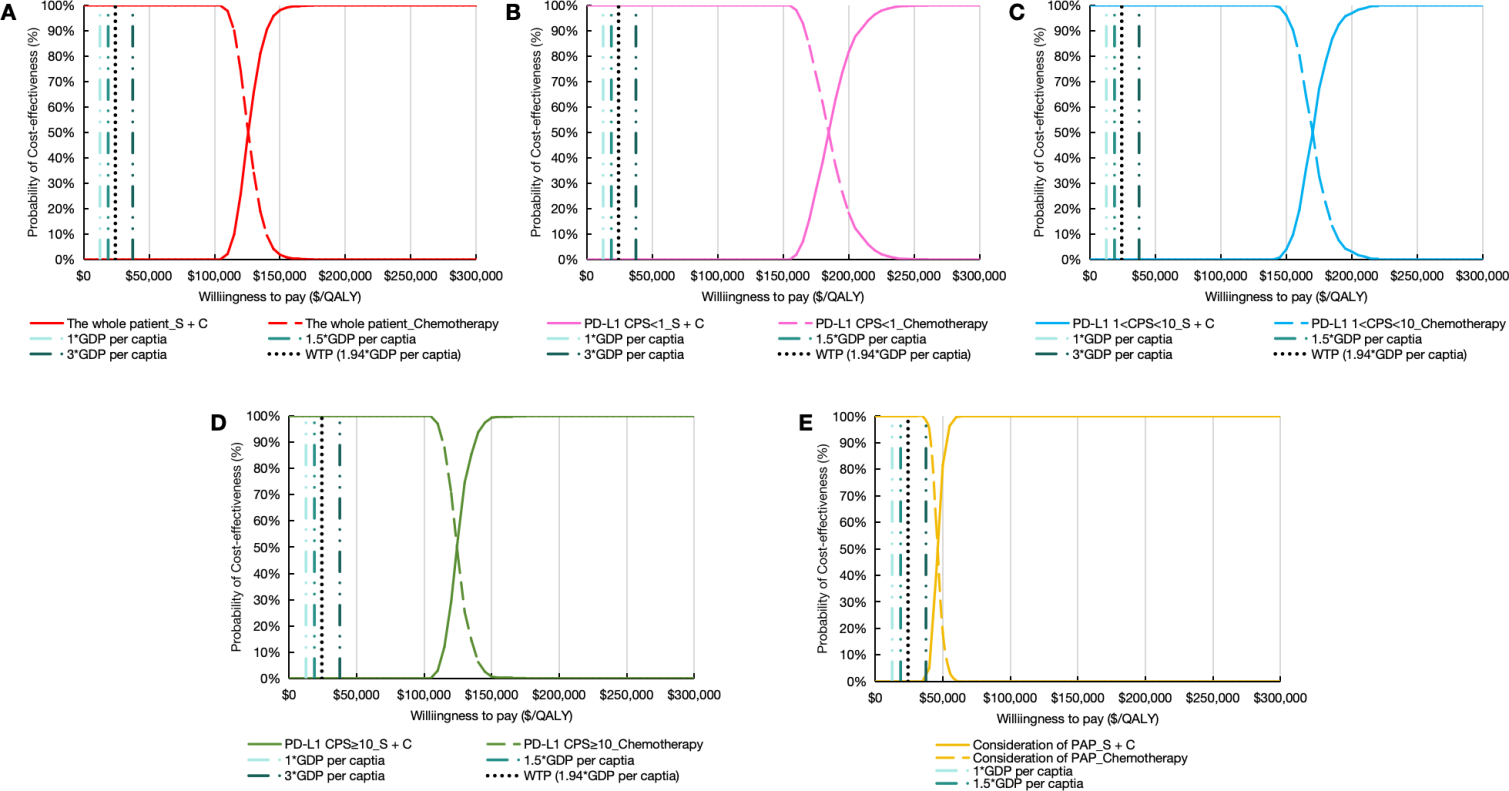
Cost-effectiveness acceptability curve. **(A)** the whole patient. **(B)** patients with PD-L1 CPS < 1. **(C)** patients with PD-L1 1 < CPS < 10. **(D)** patients with PD-L1 CPS ≥ 10. **(E)** Consideration of PAP. PD-L1 indicates programmed death ligand-1; CPS, combined positive score; S + C, sugemalimab plus chemotherapy; GDP, gross domestic product; WTP, willingness to pay; QALYs, quality-adjusted life years; PAP, patient assistance program.

PSA demonstrated limited cost-effectiveness of sugemalimab combination therapy. The Monte Carlo simulations revealed all scenario outcomes in the northeast quadrant of the cost-effectiveness plane, exceeding the predefined WTP threshold (**Figure 4**). This pattern persisted across population subgroups, with the CEAC indicating zero probability of the intervention being cost-effective through 1,000 iterations (**Figures 5A–E**).

#### 3.2.3 ICERs based on different parameters of health utility value

Scenario analyses evaluating health utility parameters revealed consistent patterns in ICERs. All sensitivity scenarios maintained the rank order of ICER magnitudes regardless of the utility weights assigned to PFS and PD states. The intervention’s ICER values systematically exceeded the predefined WTP threshold across parameter combinations (**eTable 2**). Notably, variations in utility parameters failed to produce any scenario where combination therapy became economically favorable compared to chemotherapy alone.

### 3.3 Price cap simulations for sugemalimab

Given sugemalimab’s exclusion from China’s NRDL, price cap simulations were conducted to identify economically viable price points under China’s WTP framework for end-stage conditions (45).

**Figure 6:**
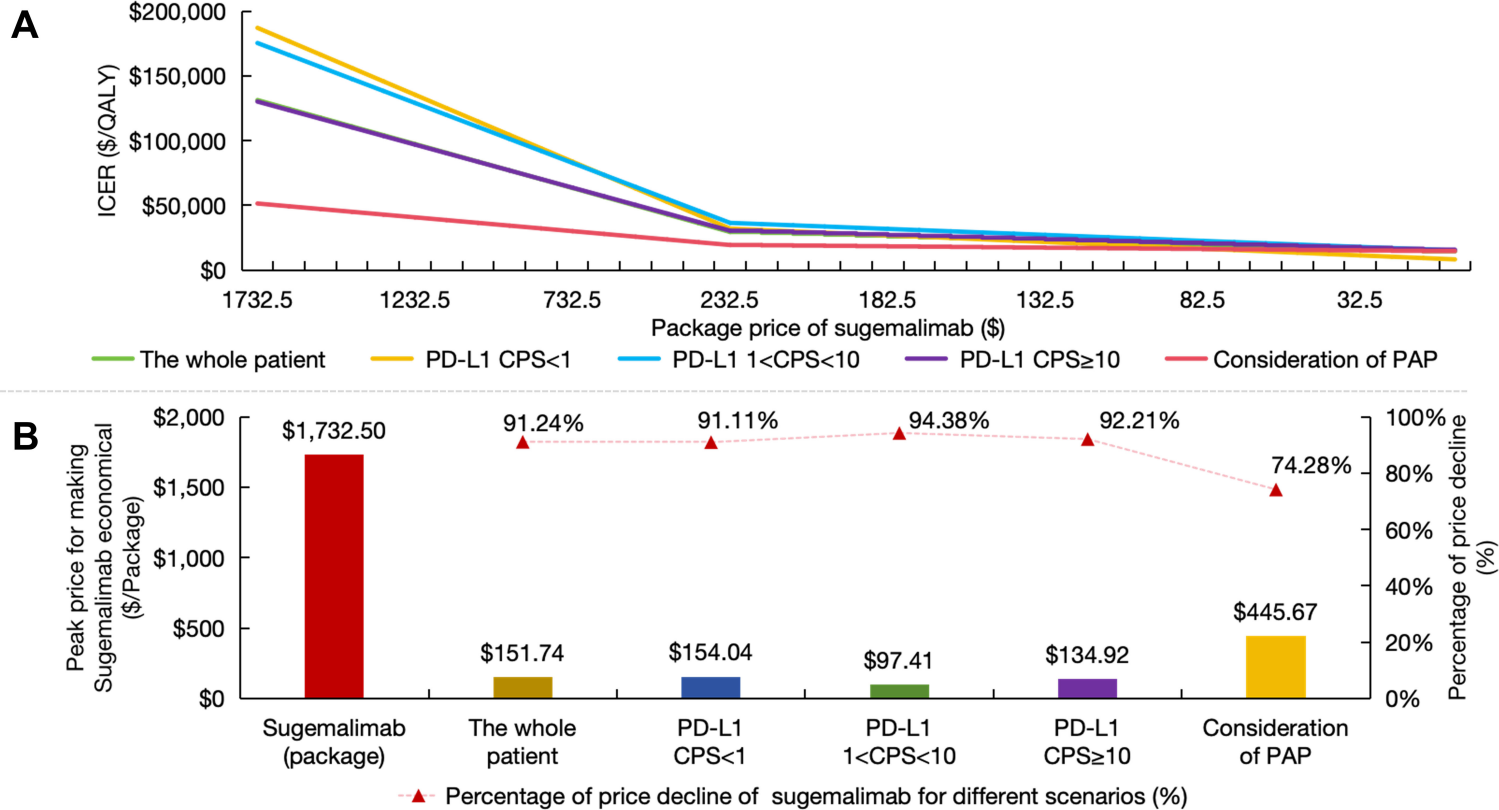
Price caps for sugemalimab. **(A)** The ICER variation curve with the decline in the package price of sugemalimab; **(B)** Price caps and reduction range that render sugemalimab economically viable under different scenarios. PD-L1 indicates programmed death ligand-1; CPS, combined positive score; PAP, patient assistance program; ICER, incremental cost-effectiveness ratio.

Price cap simulations demonstrated relationships between drug cost reductions and cost-effectiveness probabilities. The intervention only achieved cost-effectiveness when sugemalimab package pricing was reduced by 91.24% from baseline ($ 1732.50 to $ 151.74) in the overall population. Patient subgroups stratified by PD-L1 expression levels required greater price concessions, with required reductions ranging from 91.11% to 94.38% across CPS categories. Complementary analysis incorporating PAP identified a separate viability threshold at $ 445.67 (74.28% reduction), though this remained substantially above conventional cost-effectiveness benchmarks (**Figure 6**).

These findings collectively indicate that NRDL inclusion would necessitate unprecedented price restructuring for sugemalimab. Achieving the identified price thresholds could theoretically improve immunotherapy accessibility for ESCC patients, though the required magnitude of price adjustments (exceeding 90% in most scenarios) presents substantial implementation challenges for manufacturers and payers.

## 4 Discussion

The current therapeutic landscape for advanced ESCC in China remains dominated by conventional chemotherapy, radiotherapy, and surgical interventions, particularly for late-stage patients ineligible for curative resection (30). While these established modalities provide modest survival benefits, their clinical utility is constrained by suboptimal efficacy profiles and significant quality-of-life compromises. The recent introduction of immunotherapeutic agents like sugemalimab represents a paradigm shift in treatment approaches, though systemic barriers persist in translating clinical trial efficacy into real-world accessibility. Geographic disparities in healthcare resource distribution create pronounced inequities, while economic constraints further exacerbate this divide, as high out-of-pocket costs frequently lead to treatment discontinuation or suboptimal dosing regimens, particularly under China’s evolving universal healthcare coverage framework.

This study conducts a cost-effectiveness evaluation of sugemalimab-chemotherapy as first-line therapy for advanced ESCC using a PSM. The base-case analysis demonstrated an incremental gain of 0.336 QALYs at an additional cost of $ 44,182.03 compared to chemotherapy alone, with the regimen failing to meet the predefined WTP threshold. This result aligns with a prior pharmacoeconomic study (46), which concluded that the intervention lacks economic viability in the whole ESCC patients. Building upon this consensus, our analysis extends the evidence through three critical dimensions. First, CPS-stratified subgroup analyses systematically confirmed economic infeasibility across all PD-L1 expression levels. Second, PAP scenarios marginally decreased incremental costs, but failed to present cost-effectiveness advantages. Third, price cap simulations identified a 91.2% reduction requirement for sugemalimab’s current price to align with China’s pharmacoeconomic benchmarks.

The OWSA identified sugemalimab’s unit cost as the most predominant driver of ICER variability. In parallel, PSA incorporating cohort-wide, CPS-subgroup, and PAP scenarios demonstrated complete alignment with base-case outcomes. Monte Carlo simulations yielded zero ICER pairs within the cost-effective quadrant (ICER < the predefined WTP threshold), with corresponding CEACs also showing 0% probability of economic viability. These deterministic and stochastic analyses collectively indicate that no examined population subset or pricing modification achieves cost-effectiveness under the current pricing model. Sugemalimab has obtained regulatory approval in China for five oncologic indications, including squamous/non-squamous NSCLC and ESCC. Notably, published cost-effectiveness evaluations in NSCLC (47–53) all corroborate the systemic misalignment between its clinical benefits and current pricing across indications. This recurrent pharmacoeconomic paradox might arises from dual mechanisms: 1) The multi-indication development paradigm, while enabling R&D cost amortization through expanded therapeutic applications, paradoxically amplifies payer demands for volume-based price concessions during NRDL negotiations; 2) China’s value assessment framework continues to prioritize cost containment over therapeutic innovation for multi-indication biologics. Consequently, implementing an indication-stratified pricing framework—establishing disease-specific price thresholds coupled with volume-price agreements—may constitute the optimal strategic pathway to reconcile manufacturer returns with healthcare system sustainability.

Notably, several methodological assumptions inherent to our analytical framework may constrain the interpretative validity of the findings, introducing potential biases and uncertainties. Firstly, although the GEMSTONE-304 trial was conducted in China with domestic investigators, the absence of explicit regional recruitment documentation prevents confirmation of complete geographical representativeness between trial participants and broader ESCC populations (27). Secondly, the inherent selection bias of RCTs—enforcing restrictive eligibility criteria—may systematically overestimate therapeutic efficacy while underestimating safety risks prevalent in heterogeneous patient populations, as clinically significant comorbidities frequently excluded from RCT protocols routinely influence treatment feasibility and outcomes in real-world clinical settings. Thirdly, the exclusive focus on direct medical costs systematically underestimates societal economic burden by omitting direct non-medical costs and indirect costs (such as caregiving expenses, productivity losses, and ancillary medical supplies, etc) (32). Fourthly, the AE analysis framework exhibits dual constraints: it only incorporates grade ≥3 AEs with >3% incidence (nausea, cytopenias, etc.), excluding low-grade toxicities, while deriving management costs from literature sources rather than real-world claims data; and the treatment cost data of AEs in this study comes from the published relevant literature rather than real-world data, which cannot represent the true medical economic level in China (32). Fifthly, the binary treatment comparison (sugemalimab-chemo vs chemo alone) oversimplifies clinical decision-making. Only comparing the outcomes of two interventions may not provide sufficient guidance for clinicians faced with multiple treatment options in real-world clinical settings (54). Despite these constraints, the model’s adherence to trial-derived survival parameters maintains internal validity for the evaluated treatment paradigm. Through structural validation of the partitioned survival architecture, we optimized extrapolation robustness while constraining assumptions within evidence boundaries, thereby enabling contextually optimized cost-effectiveness inference that aligns with clinical decision-making frameworks.

## 5 Conclusions

This study reveals that sugemalimab-chemotherapy, while providing survival benefits, fails to demonstrate cost-effectiveness advantages in China’s healthcare context for advanced ESCC—consistent across overall and subgroup analyses. Patient assistance programs mitigate but cannot overcome the regimen’s economic barriers. Until further clinical evidence establishes superior safety-efficacy advantages of sugemalimab-chemotherapy, strategic price realignment remains the essential mechanism to balance therapeutic innovation with equitable accessibility, thereby aligning with China’s healthcare affordability priorities.

## Data Availability

The original contributions presented in the study are included in the article/**Supplementary Material**. Further inquiries can be directed to the corresponding author.
