# Supplementary material for: Sugemalimab plus chemotherapy vs. chemotherapy for treatment of Chinese patients with esophageal squamous cell carcinoma: a cost effectiveness analysis to inform decision making
Source: Front Oncol. 2025 Jun 5;15:1459695. doi: 10.3389/fonc.2025.1459695 (PMC12176590; doi:10.3389/fonc.2025.1459695)
Supplement: Supplementary file 1 [file Table1.docx]

**eTable 1. Details of fitting function and parameters of survival curve of each scenario.**

| **Patients** | **Data** | **Distribution** | **Parameter 1** | **Parameter 2** | **Parameter 3** | **AIC** | **BIC** |
| --- | --- | --- | --- | --- | --- | --- | --- |
| The whole patient | Sugemalimab plus chemotherapy (PFS) | Exponential | 0.09 |  |  | 1700.57 | 1704.45 |
|  |  | Weibull | 0.03 | 1.41 |  | 1659.90 | 1667.66 |
|  |  | **Log-logistic** | **9.06** | **2.15** |  | **1633.05** | **1640.81** |
|  |  | Log-normal | 2.15 | 0.89 |  | 1654.31 | 1662.07 |
|  |  | Gompertz | 0.06 | 0.06 |  | 1690.77 | 1698.53 |
|  |  | Generalized Gamma | 2.30 | 0.75 | 0.22 | 1646.62 | 1658.26 |
|  | Sugemalimab plus chemotherapy (OS) | Exponential | 0.04 |  |  | 1461.79 | 1465.67 |
|  |  | Weibull | 0.03 | 1.09 |  | 1443.36 | 1451.13 |
|  |  | **Log-logistic** | **20.92** | **1.66** |  | **1442.43** | **1450.19** |
|  |  | Log-normal | 3.06 | 1.05 |  | 1450.93 | 1458.69 |
|  |  | Gompertz | 0.02 | 0.02 |  | 1450.11 | 1457.87 |
|  |  | Generalized Gamma | 3.34 | 0.91 | 0.79 | 1444.04 | 1455.68 |
|  | Chemotherapy (PFS) | Exponential | 0.13 |  |  | 848.43 | 851.63 |
|  |  | Weibull | 0.05 | 1.52 |  | 812.59 | 818.99 |
|  |  | **Log-logistic** | **6.61** | **2.53** |  | **791.57** | **797.97** |
|  |  | Log-normal | 1.61 | 0.74 |  | 800.10 | 806.51 |
|  |  | Gompertz | 0.06 | 0.12 |  | 840.73 | 847.14 |
|  |  | Generalized Gamma | 1.91 | 0.72 | 0.20 | 799.46 | 809.07 |
|  | Chemotherapy (OS) | Exponential | 0.05 |  |  | 804.31 | 807.51 |
|  |  | Weibull | 0.01 | 1.51 |  | 783.82 | 790.22 |
|  |  | **Log-logistic** | **16.12** | **2.00** |  | **779.32** | **785.73** |
|  |  | Log-normal | 2.79 | 0.91 |  | 781.53 | 787.94 |
|  |  | Gompertz | 0.04 | 0.03 |  | 795.27 | 801.68 |
|  |  | Generalized Gamma | 3.09 | 0.73 | 1.32 | 782.17 | 791.78 |
| PD-L1 CPS <1 | Sugemalimab plus chemotherapy (PFS) | Exponential | 0.09 |  |  | 218.64 | 220.35 |
|  |  | Weibull | 0.03 | 1.35 |  | 217.16 | 220.59 |
|  |  | **Log-logistic** | **7.92** | **2.05** |  | **212.24** | **215.66** |
|  |  | Log-normal | 2.23 | 0.91 |  | 219.32 | 222.75 |
|  |  | Gompertz | 0.06 | 0.06 |  | 220.24 | 223.67 |
|  |  | Generalized Gamma | 2.26 | 0.81 | 0.31 | 217.60 | 222.74 |
|  | Sugemalimab plus chemotherapy (OS) | Exponential | 0.03 |  |  | 187.87 | 189.59 |
|  |  | **Weibull** | **0.01** | **1.32** |  | **187.17** | **190.60** |
|  |  | Log-logistic | 19.26 | 1.79 |  | 187.59 | 191.02 |
|  |  | Log-normal | 2.94 | 1.05 |  | 187.77 | 191.19 |
|  |  | Gompertz | 0.02 | 0.03 |  | 187.87 | 191.30 |
|  |  | Generalized Gamma | 3.12 | 0.79 | 0.69 | 189.09 | 194.23 |
|  | Chemotherapy (PFS) | Exponential | 0.17 |  |  | 96.67 | 97.71 |
|  |  | Weibull | 0.04 | 1.46 |  | 94.66 | 96.75 |
|  |  | Log-logistic | 6.70 | 2.50 |  | 95.72 | 97.80 |
|  |  | **Log-normal** | **1.80** | **0.72** |  | **94.36** | **96.45** |
|  |  | Gompertz | 0.05 | 0.12 |  | 96.16 | 98.24 |
|  |  | Generalized Gamma | 1.97 | 0.86 | 0.17 | 96.13 | 99.26 |
|  | Chemotherapy (OS) | Exponential | 0.05 |  |  | 104.50 | 105.55 |
|  |  | Weibull | 0.01 | 1.47 |  | 104.31 | 106.40 |
|  |  | Log-logistic | 14.55 | 2.15 |  | 104.33 | 106.42 |
|  |  | **Log-normal** | **2.62** | **0.86** |  | **104.25** | **106.34** |
|  |  | Gompertz | 0.04 | 0.03 |  | 105.30 | 107.39 |
|  |  | Generalized Gamma | 2.94 | 0.61 | 1.04 | 106.07 | 109.20 |
| PD-L1 1 < CPS <10 | Sugemalimab plus chemotherapy (PFS) | Exponential | 0.09 |  |  | 793.87 | 796.97 |
|  |  | Weibull | 0.04 | 1.30 |  | 784.47 | 790.66 |
|  |  | **Log-logistic** | **8.21** | **1.82** |  | **770.10** | **776.29** |
|  |  | Log-normal | 2.20 | 0.87 |  | 772.26 | 778.45 |
|  |  | Gompertz | 0.06 | 0.05 |  | 794.66 | 800.85 |
|  |  | Generalized Gamma | 2.20 | 0.89 | 0.32 | 774.14 | 783.42 |
|  | Sugemalimab plus chemotherapy (OS) | Exponential | 0.04 |  |  | 714.95 | 718.04 |
|  |  | Weibull | 0.01 | 1.34 |  | 710.84 | 716.26 |
|  |  | **Log-logistic** | **18.14** | **1.59** |  | **710.07** | **717.03** |
|  |  | Log-normal | 2.89 | 0.92 |  | 714.29 | 720.47 |
|  |  | Gompertz | 0.02 | 0.03 |  | 713.03 | 719.21 |
|  |  | Generalized Gamma | 3.10 | 0.83 | 0.72 | 711.83 | 721.11 |
|  | Chemotherapy (PFS) | Exponential | 0.13 |  |  | 400.77 | 403.19 |
|  |  | Weibull | 0.05 | 1.45 |  | 383.77 | 388.60 |
|  |  | **Log-logistic** | **7.32** | **2.20** |  | **364.43** | **369.27** |
|  |  | Log-normal | 2.00 | 0.72 |  | 367.46 | 372.30 |
|  |  | Gompertz | 0.06 | 0.11 |  | 399.00 | 403.84 |
|  |  | Generalized Gamma | 1.93 | 0.73 | 0.19 | 368.93 | 376.18 |
|  | Chemotherapy (OS) | Exponential | 0.04 |  |  | 358.04 | 360.46 |
|  |  | Weibull | 0.01 | 1.42 |  | 347.43 | 352.27 |
|  |  | Log-logistic | 16.55 | 2.00 |  | 342.48 | 347.32 |
|  |  | **Log-normal** | **2.81** | **0.83** |  | **340.86** | **345.70** |
|  |  | Gompertz | 0.03 | 0.03 |  | 355.26 | 360.09 |
|  |  | Generalized Gamma | 2.92 | 1.01 | 0.51 | 341.87 | 349.13 |
| PD-L1 CPS ≥ 10 | Sugemalimab plus chemotherapy (PFS) | Exponential | 0.09 |  |  | 715.82 | 718.86 |
|  |  | Weibull | 0.01 | 1.82 |  | 680.74 | 686.82 |
|  |  | **Log-logistic** | **10.00** | **2.50** |  | **672.95** | **679.02** |
|  |  | Log-normal | 2.23 | 0.71 |  | 677.41 | 683.48 |
|  |  | Gompertz | 0.08 | 0.04 |  | 697.31 | 703.39 |
|  |  | Generalized Gamma | 2.44 | 0.70 | 0.25 | 677.20 | 686.31 |
|  | Sugemalimab plus chemotherapy (OS) | Exponential | 0.03 |  |  | 564.40 | 567.44 |
|  |  | Weibull | 0.01 | 1.58 |  | 553.27 | 559.35 |
|  |  | **Log-logistic** | **22.51** | **1.90** |  | **552.15** | **558.22** |
|  |  | Log-normal | 3.11 | 0.94 |  | 554.74 | 560.81 |
|  |  | Gompertz | 0.04 | 0.02 |  | 558.51 | 564.58 |
|  |  | Generalized Gamma | 3.44 | 0.68 | 1.10 | 554.62 | 563.73 |
|  | Chemotherapy (PFS) | Exponential | 0.15 |  |  | 358.91 | 361.28 |
|  |  | Weibull | 0.05 | 1.50 |  | 344.22 | 348.96 |
|  |  | **Log-logistic** | **6.62** | **2.54** |  | **338.32** | **343.06** |
|  |  | Log-normal | 1.70 | 0.87 |  | 340.64 | 345.38 |
|  |  | Gompertz | 0.06 | 0.12 |  | 355.81 | 360.55 |
|  |  | Generalized Gamma | 1.93 | 0.80 | 0.21 | 341.43 | 348.54 |
|  | Chemotherapy (OS) | Exponential | 0.04 |  |  | 345.74 | 348.09 |
|  |  | Weibull | 0.01 | 1.45 |  | 339.61 | 344.32 |
|  |  | **Log-logistic** | **14.96** | **1.90** |  | **339.34** | **344.06** |
|  |  | Log-normal | 2.77 | 0.92 |  | 341.01 | 345.72 |
|  |  | Gompertz | 0.04 | 0.03 |  | 342.72 | 347.43 |
|  |  | Generalized Gamma | 3.07 | 0.73 | 1.10 | 341.23 | 348.30 |

Abbreviations: CPS, combined positive score; PFS, progression-free survival; OS, overall survival.
