# Supplementary material for: Sugemalimab plus chemotherapy vs. chemotherapy for treatment of Chinese patients with esophageal squamous cell carcinoma: a cost effectiveness analysis to inform decision making
Source: Front Oncol. 2025 Jun 5;15:1459695. doi: 10.3389/fonc.2025.1459695 (PMC12176590; doi:10.3389/fonc.2025.1459695)
Supplement: Supplementary file 2 [file Table2.docx]

**eTable 2. ICERs with different parameters of health utility values of PFS and PD state.**

| References | Health utility value | ICER ($ / QALY) | |
| --- | --- | --- | --- |
| liu et al. [42] | PFS: 0.750 PD: 0.600 | The whole patient | 131,544.70 |
|  |  | PD-L1 CPS < 1 | 187,421.63 |
|  |  | PD-L1 1 < CPS <10 | 175,689.56 |
|  |  | PD-L1 CPS > 10 | 130,349.21 |
|  |  | Consideration of PAP | 51,454.12 |
| Li et al. [49] | PFS: 0.680 PD: 0.420 | The whole patient | 162,106.13 |
|  |  | PD-L1 CPS < 1 | 215,949.23 |
|  |  | PD-L1 1 < CPS <10 | 217,744.72 |
|  |  | PD-L1 CPS > 10 | 161,478.51 |
|  |  | Consideration of PAP | 63,408.31 |
| Zhang et al. [50] | PFS: 0.741 PD: 0.581 | The whole patient | 134,374.46 |
|  |  | PD-L1 cps < 1 | 190,409.03 |
|  |  | PD-L1 1 < CPS <10 | 179,549.46 |
|  |  | PD-L1 CPS > 10 | 133,208.28 |
|  |  | Consideration of PAP | 52,560.99 |
